# What Does Heterosexuality Mean? Same-Sex Attraction, Behaviors, and Discomfort Among Self-identified Heterosexual Young Adults from Spain

**DOI:** 10.1007/s10508-022-02315-6

**Published:** 2022-09-06

**Authors:** Juan E. Nebot-Garcia, Cristina Giménez-García, Marta García-Barba, María Dolores Gil-Llario, Rafael Ballester-Arnal

**Affiliations:** 1 Department of Clinical and Basic Psychology and Psychobiology, Universitat Jaume I, Avda. Vicent Sos Baynat s/n, 12071 Castellón de la Plana, Spain; 2 Department of Developmental and Educational Psychology, Universitat de València, Valencia, Spain

**Keywords:** Same-sex experiences, Behavioral intention, Heterosexuality, Young adults, Gender differences, Sexual orientation

## Abstract

Sexuality has been censored by Spanish culture, where legal progress in the form of new policies about sexual diversity rights has not been followed by lower levels of social discrimination. This has affected sexual development across the lifespan for both sexual minorities and heterosexual people who experience their sexuality outside of heteronormativity. However, the literature has regularly excluded the possible consequences of the experience of sexuality within prescriptive categories, particularly in heterosexual people. This study delves deeper into the same-sex experiences of heterosexual women and men and the discomfort they may feel toward such experiences in Spain. A total of 2900 young Spanish people who self-identified as heterosexual completed a questionnaire on sexual diversity and experiences related to sexual orientation. The mean age was 24.22 years (SD = 5.71), 71.1% were women, and 28.9% were men. More heterosexual women than heterosexual men reported having had same-sex attraction, fantasies, desires, and behavioral intentions. However, these men and women reported having similar levels of same-sex sexual intercourse. The discomfort level experienced was higher among heterosexual men and younger people, suggesting the possibility that traditional norms restrict behavioral expression of sexuality among these individuals. At least in the Spanish context, these gender differences should be taken into account to adjust all psychological and educational interventions in the future to improve inclusive sexual attitudes and the treatment of discomfort with sexual orientation. Following these results, research on sexual diversity should focus on young heterosexual people who have different same-sex experiences according to gender differences.

## Introduction

A group may be formed when several people share certain defining characteristics. People need to compare themselves with others to classify themselves and feel part of a group. When the characteristics of the group are clear and a person complies with the prototypes, the person can be defined as such and categorized within the group. However, when the patterns are not clear or are not met, uncertainty and discomfort may appear because the person does not feel as though they are part of the group (Hogg, 2004).

This mechanism can be observed in awareness of sexual orientation. In this sense, when there is dissonance between what heterosexual people experience and what social norms dictate regarding heterosexuality, discomfort may appear (Krueger et al., 2018). This discomfort might be heightened by heterosexism and monosexism. First, heterosexism is a social structure that suggests that heterosexuality is the only morally acceptable sexual orientation and implies structural oppression against people with other orientations (Eisner, 2013). Second, monosexism considers that all people are attracted to one gender, which privileges to heterosexual and homosexual people and systematically punishes people who are attracted to more than one gender (Eisner, 2013; Hayfield, 2020).

These beliefs and attitudes would affect heterosexual people with same-sex experiences, who see their sexuality as limited and perceive their same-sex experiences as something dangerous that would cause a loss of their privileges as heterosexuals (Eisner, 2013). In addition, these discordant experiences could create uncertainty about sexual self-concept of heterosexual people. This ambiguity in their sexual orientation, their doubts, and not complying with social norms around heterosexuality could be significant factors in the perceived discomfort of heterosexual people. The perceived discomfort is a variable that is often used in clinical contexts (Kalfon et al., 2010; Lin et al., 2010) and in studies focusing on the lesbian, gay, bisexual, transgender, or intersex (LGBTI) individuals (Rose et al., 2017; Scroggs et al., 2020). Several studies have found that heterosexual individuals with same-sex experiences and people who question their sexual orientation perceive higher levels of emotional impacts. Krueger et al. (2018) analyzed the data of 14,216 young adults from the US and found that heterosexual people with discordant same-sex attractions or behaviors report having more depressive symptoms than do those with concordant ones. However, among 263 university students from the US, Morales-Knight and Hope (2012) did not find any statistically significant differences between the emotional well-being of heterosexuals who had had same-sex experiences and that of those who had not. Other studies have found that people who have doubts or have questioned their sexual orientation report lower levels of psychological well-being and poorer mental health. Among 7th- and 8th-grade students from the US, Birkett et al. (2009) found that those who questioned their sexual orientation report more bullying and homophobic victimization and experience more intense feelings of depression and suicidal tendencies than do heterosexual or LGB students. In another study with 2513 North American youth aged between 14 and 24 years, girls who questioned their sexual orientation report having more intense symptoms of depression, anxiety, and traumatic distress than heterosexual females, although men do not show statistically significant differences (Shearer et al., 2016).

The impact of these beliefs on personal identity would be associated with the social reference group and the cultural context in which one lives. Different populations and cultures have not shown the same level of tolerance and permissiveness toward homosexuality and gender-sexual scripts (Kite et al., 2019; Petersen & Hyde, 2011). In particular, Spanish culture has traditionally placed limits on sexuality. As a consequence, the legal progress that has been made in the form of new policies about sexual diversity rights has not been followed by a lower level of discrimination that has usually been based on marianismo and religious tradition. According to this assumption, people who have been assigned as men or women at birth must assume certain roles framed in machismo and femininity, monogamous heterosexual relationships, and romantic love (Giménez-García et al., 2020). Otherwise, people will be socially unpopular and will be marked as being outside the norm.

These beliefs were reinforced between 1939 and 1976 when Spain was submerged in a dictatorship. During that time, there was a legal and cultural restriction in terms of gender and sexuality (Santos, 2013), which was based on the Catholic religion premises that establish the legal and morally acceptable conceptions about the family and sexuality. Thus, there was marked sexism, and sexual relations were allowed only within marriage and for procreation. In this context, homosexuality was legally prosecuted (Pichardo, 2011). From the Spanish transition to the present day, several feminist and sexual liberation movements have been fighting for legal changes such as the right to divorce or abortion. Consequently, Spain is at the same time the fourth country to legalize same-sex marriage and adoption in the world (International Lesbian, Gay, Bisexual, Trans and Intersex Association, 2020) and a place where there is still discrimination against LGBTI individuals (Rault, 2020). In fact, there is strongly extremist opposition against the rights of the LGBTI collective by some parties in the Congress of Deputies (Abou-Chadi & Finnigan, 2019; Rama et al., 2021; Rodríguez-Temiño & Almansa-Sánchez, 2021). This situation is in line with results of a 2019 European survey that revealed the following: 8% of Spanish LGBTI people (aged 15 years and over) had experienced physical and/or sexual attacks in the preceding 5 years because of their sexual orientation; 41% had experienced harassment in the preceding 12 months because of their sexual orientation; and 81%, at some point, had avoided holding hands with their same-sex partner in public for fear of being assaulted, threatened, or harassed (European Union Agency for Fundamental Rights, 2020).

These conservative beliefs could limit the socialization and the experience of sexuality among the Spanish population. However, only a few studies from Spain have focused on this area. Regarding the experience of sexual orientation, two studies among Spanish youth of different sexual orientations found that 19.1–28.2% of men and 10.4–24.6% of women identified as nonheterosexual (Ballester-Arnal & Gil-Llario, 2016; Nebot-Garcia et al., 2020). However, higher percentages of people with same-sex interests and desires were found. In another study that focused on heterosexual Spanish youth, 28.4% of women and 18.5% of men reported having had some degree of same-sex sexual attraction, and 3.6% of women and 4.6% of men reported having had same-sex intercourse. Compared to heterosexual men, heterosexual women are more willing to engage in same-sex sexual practices in the future (Nebot-Garcia et al., 2018).

These results show that gender can also play an important role in the experience of sexual orientation in Spanish population (Gil-Llario et al., 2017; Nebot-Garcia et al., 2018), similar to other populations such as North American people. Copen et al. (2016) found that 12.6% of heterosexual women and 2.8% of heterosexual men in North America, aged between 18 and 44 years, reported having ever had some type of same-sex sexual contact. Among university students aged between 18 and 33 years from the US, 79% and 43% of heterosexual women and men, respectively, reported having had at least some same-sex attraction, while 52% and 22% of heterosexual women and men, respectively, reported having had some same-sex fantasy (Vrangalova & Savin-Williams, 2010). Of those who were sexually experienced, 14% and 4% of heterosexual women and men, respectively, reported having had same-sex partners. In line with the literature (Gates, 2011; Petersen & Hyde, 2011), women are more likely to identify as bisexual and men as homosexual, although heterosexuality is more common among both. The Kinsey Scale includes a broad range of possibilities ranging from “exclusively heterosexual” to “exclusively homosexual” (Kinsey et al., 1948). On this scale, women have reported more intermediate positions than men, such as nonexclusive attraction to one sex (Copen et al., 2016). Similarly, women also manifest higher levels of same-sex attraction, fantasies, and sexual behaviors, despite identifying as heterosexual (Copen et al., 2016; Morales-Knight & Hope, 2012; Vrangalova & Savin-Williams, 2010, 2012). In this sense, Diamond (2008) found that, regardless of sexual orientation, women present greater variability and fluidity in their sexuality, which changes over time than men. Heterosexual women also show greater tolerance and a more positive attitude toward homosexuality than heterosexual men (Petersen & Hyde, 2011).

Despite these important findings, Spanish studies have not considered the older population and particular variables to be as relevant as the discomfort they felt after experiencing same-sex behaviors after they had self-identified as heterosexual. Two of the few studies worldwide that included a variable on emotional well-being in heterosexual people with same-sex experiences were carried out in the US. First, in Morales-Knight and Hope (2012), sexuality-related self-esteem, positive and negative affect, and social anxiety were examined. Heterosexuals who had reported having had some same-sex experience and those who had not were compared. However, the sample focused on university students, who were grouped into a single category of heterosexual individuals regardless of the type of same-sex attraction, fantasies, or behaviors. Second, Krueger et al. (2018) assessed differences in perceived stress and in depressive symptoms between heterosexuals with and without same-sex experiences. This study also focused on a very specific age group (24 to 34 years) and did not differentiate between same-sex attraction and behaviors.

To address this gap in knowledge, the current study analyzed the same-sex experiences of Spanish heterosexual young adults (18 to 40 years). In particular, in addition to same-sex attraction and the intention to perform different same-sex behaviors, this study evaluated different same-sex experiences. Second, this study evaluated the subsequent discomfort experienced after performing each of these same-sex behaviors in the Spanish context. The study aimed to explain the general discomfort experienced with one’s sexual orientation among Spanish people.

Accordingly, two hypotheses were formulated and tested: (H1) Spanish heterosexual women will show greater levels of same-sex experiences (same-sex attraction, behavioral intention to carry out same-sex behaviors, and more same-sex aesthetic-erotic experiences) than those of Spanish heterosexual men, while (H2) Spanish heterosexual men will experience more discomfort than Spanish heterosexual women for having had same-sex experiences. We also formulated a research question (RQ1): What social and demographic variables and same-sex aesthetic-erotic experiences are related to discomfort with one’s sexual orientation?

## Method

### Participants and Procedure

This study is part of a larger project on sexual diversity and experiences related to the development of sexual orientation. To disseminate this research and obtain a wide Spanish sample, from January to March 2017, we announced information on this study about sexual orientation in general groups via social networking platforms.

When the participants clicked on the advertisement, they accessed a screen in which they were informed of the anonymous, voluntary, and confidential nature of this study and were asked to provide their informed consent. After they gave their informed consent, they were free to answer the online questionnaire. This study was approved by the Ethics Committee of the University Jaume I of Castellón (Spain) and followed the ethical principles of the Declaration of Helsinki.

Through convenience sampling, 4564 responses were collected. Eventually, 2900 people who met the inclusion criteria participated. These criteria were as follows: heterosexual self-identification (*n* = 3420), age over 18 years at the time of study (*n* = 3379), and residence in Spain (*n* = 3257). We eliminated a few participants who were aged over 40 years (*n* = 357) owing to both the great dispersion in age and the possible bias their inclusion could produce.

A total of 2900 people participated in the study. Of these, 71.1% were women and 28.9% were men, with an average age of 24.22 years (SD = 5.71). All participants identified as heterosexual. Most were atheists or agnostics, had progressive political views, had higher education, and came from urban areas. Gender differences in sociodemographic variables had a weak and small effect size (see Table 1). Participants came from different parts of Spain. The most prominently represented areas were Alicante (16%), Castelló (7.3%), Madrid (7%), Salamanca (6.1%), Las Palmas (5%), Pontevedra (4.5%), Balearic Islands (4.4%), Valencia (3.8%), and Barcelona (3.7%).

**Table 1:** Main sociodemographic characteristics of participants

Variables	Men	Women	*t*	*df*	*p* values	Effect size
	*M* (SD)	*M* (SD)				
Age	25.20 (6.16)	23.82 (5.48)	− 5.64	1405	< .001	*d* = .24

	%	%	*χ*^*2*^	*df*	*p* values	Effect size
Religious beliefs						
Practicing believer	7.2	5.9	12.31	2	.002	*V* = .065
Nonpracticing believer	29.2	35.9				
Atheist or agnostic	63.6	58.2				
Level of education						
Without studies	1	0.1	51.55	6	< .001	*V* = .133
Primary	3.3	1.6				
Secondary	22.7	16.5				
Vocational training	16.8	15				
Diploma	3.6	3.6				
Bachelor/degree	44.3	55.5				
Master/doctorate	8.3	7.7				
Place of residence during childhood/adolescence						
Urban coast	31.7	31.7	1.50	3	.682	*V* = .023
Urban inland	39.4	37.4				
Rural coast	6.1	6.6				
Rural inland	22.8	24.3				
Political ideology						
Conservative	10	4.3	43.69	3	< .001	*V* = .123
Center	22.2	19.4				
Progressive	42.4	44.7				
Indifferent	25.4	31.6				

### Measures

A questionnaire about sexual orientation life experience was given to the participants. This questionnaire was developed to comprehensively evaluate the experience of sexual orientation according to 94 items that were grouped into 14 scales: aesthetic-erotic and sexual experiences with the same and the opposite sex and associated discomfort; past, present, and ideal sexual experiences (such as romantic and sexual attraction, sexual behavior and fantasies, etc.); behavioral intentions with the same and opposite sex; bullying and violence suffered; and experiences related to sexual orientation. Sociodemographic data were also collected. For this study, a total of 23 items were analyzed using Likert-type scales, and dichotomous or single-choice questions were grouped as follows.

#### Same-Sex Experiences

Following the appreciation of sexual orientation as a continuum (Kinsey et al., 1948) with different dimensions (Klein, 1978) and considering that behavioral intentions may predict future behaviors (Ajzen, 1991), this study measured the following.

##### Sexual Orientation Identification

This item asked about sexual orientation. Participants had to use one of the following labels to self-identify: “heterosexual,” “homosexual,” “bisexual,” “asexual,” or “other.”

##### Sexual Attraction

Drawing from Kinsey et al. (1948), this item asked, “Which of the following statements describes who you feel sexually attracted to?” The participants had to answer this question on a Likert-type scale that ranged from 1 = “I am attracted only to the other sex” to 7 = “I am attracted only to my own sex.” A third option was provided to asexual people: “I do not feel attracted to any sex.”

##### Behavioral Intention

Eight items evaluated the intention of different behaviors. Six were aimed at the entire population (kisses on the lips, caressing and hugging in the nude, masturbating the other person, being masturbated, performing oral sex on another person, and receiving oral sex), and two others were addressed only to men (penetrating and being penetrated anally). Another question asked whether they would be willing to do these things with same-sex people, and the response options were “no way,” “only if the other person is attractive,” “only if the other person is trustworthy,” and “easily.” This scale had an internal consistency of .90.

##### Gradation of Aesthetic-Erotic Experiences Related to Same-Sex People

This scale comprised six items and was used to measure the appreciation of aesthetic beauty or erotic experience related to same-sex people. The following “yes” or “no” questions were asked:

- Have you ever considered someone of the same sex to be good-looking (Item 1) or attractive (Item 2)?
- Have you had erotic dreams (Item 3), fantasies (Item 4), or the desire to have sex (including sexual touching and/or masturbation) with same-sex people (Item 5)?
- Have you had sexual intercourse (including touching and/or masturbation) with people of the same sex (Item 6)?

This scale had an internal consistency of .64.

#### Perceived Discomfort

Perceived discomfort is a concrete aspect of negative emotional impacts, which is relevant and widely used in the clinical context (Kalfon et al., 2010; Lin et al., 2010; Milot-Lapointe et al., 2019). For this reason, in this part, the related instrument evaluated the participants’ perception of discomfort in each same-sex experience and with their sexual orientation.

##### Perceived Discomfort Around Aesthetic-Erotic Experiences Related to Same-Sex People

When the question on same-sex aesthetic-erotic experiences was answered in the affirmative, the participants were asked to indicate whether the fact of having considered a person of the same sex beautiful or attractive or of having had dreams, fantasies, desires, or sexual relations with such a person had generated discomfort. They had to choose between “Yes” and “No.” This scale had an internal consistency of .73.

##### General Discomfort with One’s Sexual Orientation

The level of discomfort was assessed through the following question: “Over the years, how much discomfort have you felt with respect to your sexual orientation?” The response options were “none,” “a little,” “quite a lot,” and “a lot.”

### Statistical Analysis

Different analyses were carried out using the statistical program SPSS version 25. First, descriptive analyses were performed to evaluate the percentages of the different variables, and chi-square tests were used to examine whether there were any differences between men and women. To facilitate the analysis and interpretation of the results, the intermediate responses on the Behavioral Intention scale (“only if he or she is attractive” and “only if he or she is trustworthy”) were grouped and recoded as “it depends.”

An ordinal logistic regression was performed to analyze the percentage of discomfort with sexual orientation that was explained by the different aesthetic-erotic experiences with same-sex people and by different sociodemographic variables. However, as “quite a lot” and “a lot” in the discomfort variable were selected by less than 1% of the participants and the model presented an overall weak fit, we performed a binary logistic regression. The responses to the question regarding discomfort with one’s sexual orientation were dichotomized as follows: “0 = nothing” and “1 = a little” (combining “a little,” “quite a lot,” and “a lot”). Similarly, religious beliefs (“0 = nonbeliever,”, i.e., atheist or agnostic and “1 = believer,”, i.e., practicing and nonpracticing), type of locality (“0 = urban,”, i.e., urban coastal and inland, and “1 = rural,”, i.e., rural coastal and inland), and political ideology (0 = nonconservative, i.e., center, indifferent, and progressive, and “1 = conservative”) were also dichotomized to be included.

## Results

### Analyses of Same-Sex Experiences

#### Sexual Attraction

Sexual attraction was measured using the Kinsey Scale, wherein 68.5% and 86.8% of women and men who self-identified as heterosexual, respectively, reported feeling attraction only for the other sex. The remaining 31.5% and 13.2% of heterosexual women and men, respectively, did not exhibit such exclusive attraction (see Table 2). These differences were statistically significant (*χ*^*2*^ = 114.77; *df* = 7; *p* < .001).

**Table 2:** Gender differences in sexual attraction

Variables	Men (%)	Women (%)
I feel attraction only for the other sex	86.8	68.5
I am almost always attracted to the other sex and very rarely to my own sex	11.6	28.1
I am slightly more attracted to the other sex than toward my own sex	1	2.1
I feel equal attraction for either sex	0.2	0
I am slightly more attracted to my own sex than toward the other sex	0.1	0
I am almost always attracted to my own sex and very rarely to the other sex	0.1	0.6
I feel attraction only for my own sex	0.2	0.5
I am not attracted to either sex	0	0.2

#### Behavioral Intention

There were significant differences between heterosexual men and women in terms of the intention to perform sexual behaviors with same-sex people (see Table 3). Heterosexual women were more willing to perform such behaviors than heterosexual men. Both groups were as willing to perform “kisses on the lips”, which shows the largest significant differences between heterosexual men and women. The options of “performing oral sex on another person” and “being penetrated anally” were least preferred among heterosexual women and men, respectively.

**Table 3:** Gender differences in intentions to engage in sexual behavior among same-sex people

Variables	Men (%)	Women (%)	*χ*^*2*^	*df*	*p* values
Kisses on the lips					
No way	61.2	18.4	520.03	2	< .001
It depends	31.2	57.8			
Easily	7.6	23.8			
Nude caressing and hugging					
No way	84.5	55.9	214.19	2	< .001
It depends	14.3	38.1			
Easily	1.2	6			
Masturbating the other person					
No way	87.6	68.9	109.69	2	< .001
It depends	11.3	27.6			
Easily	1.1	3.5			
Being masturbated					
No way	83.7	63.3	116.45	2	< .001
It depends	14	31.2			
Easily	2.3	5.5			
Performing oral sex					
No way	89.6	74	87.23	2	< .001
It depends	9.6	23.2			
Easily	0.8	2.8			
Receiving oral sex					
No way	84.3	65.8	98.68	2	< .001
It depends	13.3	28.8			
Easily	2.4	5.4			
Penetrating anally^a^					
No way	89	–	–		
It depends	8.8	–			
Easily	2.2	–			
Being penetrated anally^a^					
No way	92.5	–	–		
It depends	6.6	–			
Easily	0.9	–			

The use of dashes indicates that data on these variables were not collected for women; therefore, the analyses could not be performed

^a^These variables were only evaluated in men

#### Gradation of Aesthetic-Erotic Experiences Related to Same-Sex People

Aesthetic-erotic experiences with same-sex people were more frequent among heterosexual women than among heterosexual men (see Table 4), thus presenting significant differences in all cases, except for “sexual intercourse.” For both groups, the most and least performed experience was considering same-sex people “pretty/handsome” and having sex with same-sex people, respectively. However, considering a same-sex person “attractive” showed the greatest statistically significant differences between heterosexual women and men.

**Table 4:** Differential analyses, according to gender, of aesthetic-erotic experiences related to same-sex people

	Men (%)	Women (%)	*χ*^*2*^	*df*	*p* values
Pretty/handsome	83.4	97.7	202.76	1	< .001
Attractive	66.6	92.3	307.30	1	< .001
Erotic dreams	13.9	39	172.28	1	< .001
Sexual fantasies	8.2	17.9	43.21	1	< .001
Sexual desires	11.1	26.8	84.96	1	< .001
Sexual intercourse	6.1	7.4	1.53	1	.215

### Analyses of Perceived Discomfort

#### Perceived Discomfort Around Aesthetic-Erotic Experiences Related to Same-Sex People

Gender differences were found in the discomfort of undergoing same-sex experiences, with heterosexual men scoring higher than women did (see Table 5). Considering someone of the same sex “pretty/handsome” caused the least discomfort among both heterosexual women and men. Having sexual intercourse with someone of the same sex caused the greatest discomfort, followed by same-sex erotic dreams. This item also reveals the greatest statistically significant differences between heterosexual men and women.

**Table 5:** Gender differences in the feeling of discomfort with different aesthetic-erotic experiences related to same-sex people

Variables	Men (%)	Women (%)	*χ*^*2*^	*df*	*p* values
Recognition of same-sex people as "pretty/handsome"	3.7	1.8	8.07	1	.004
Recognition of same-sex people as "attractive"	4.7	2.8	4.84	1	.028
Homoerotic dreams	35.9	16	26.92	1	< .001
Same-sex fantasies	13	5.4	5.42	1	.020
Same-sex desires	26.9	8.9	25.40	1	< .001
Same-sex sexual intercourse	54.9	17.1	27.94	1	< .001

#### Variables Related to General Discomfort with One’s Sexual Orientation

The model extracted by binary logistic regression (enter method) showed that discomfort toward one’s sexual orientation is generally related to age, level of education, gender, and erotic experiences with same-sex people (see Table 6). The model explained 14.6% of the variance. It was a statistically significant model (*χ*^*2*^ = 183.55; *df* = 12; *p* < .001) and presented a good fit overall (*χ*^*2*^ = 12.41; *df* = 8; *p* = .134).

**Table 6:** Values of the parameters of the regression model

General discomfort	*β*	SE	Wald	*df*	*p* values		95% CI	
						OR	Lower	Upper
Constant	− 3.60	1.13	10.11	1	.001	0.02		
Religious beliefs	− 0.01	0.15	0.01	1	.929	0.98	0.72	1.34
Rural residence	0.06	0.15	0.16	1	.683	1.06	0.78	1.44
Conservative	− 0.01	0.34	0.01	1	.964	0.98	0.49	1.94
Age	− 0.07	0.01	24.18	1	< .001	0.92	0.89	0.95
Education	− 0.17	0.05	11.19	1	.001	0.84	0.76	0.93
Men	0.86	0.16	26.18	1	< .001	2.37	1.70	3.31
Good-looking	2.33	1.01	5.29	1	.021	10.37	1.41	76.13
Attractive	0.68	0.31	4.78	1	.029	1.97	1.07	3.63
Dreams	0.59	0.17	12.36	1	< .001	1.81	1.30	2.53
Fantasies	0.50	0.19	6.98	1	.008	1.65	1.13	2.40
Desires	0.81	0.18	19.16	1	< .001	2.25	1.56	3.24
Sexual intercourse	0.57	0.22	6.73	1	.009	1.78	1.15	2.76

Pseudo *R* Square (Nagelkerke) = .146

The OR obtained indicates that if a heterosexual person has considered someone of the same sex good-looking, then the probability of feeling general discomfort with their sexual orientation is 10.37 times greater than if they had not considered it. If this heterosexual person is also a man (2.37) and has felt the desire to have sexual intercourse with someone of the same sex (2.25), then the probability of feeling discomfort is greater, that is, 55.29 (10.37 × 2.37 × 2.25) times more than if this heterosexual person has not considered this, has not experienced the desire to have sexual relations with someone of the same sex, and is a woman.

## Discussion

In light of these results, our findings support two main facts. First, many members of the Spanish population would have same-sex experiences despite self-identifying as heterosexual. These findings are in line with those of previous studies (Copen et al., 2016; Morales-Knight & Hope, 2012; Nebot-Garcia et al., 2018; Vrangalova & Savin-Williams, 2010, 2012) that have observed how people who self-identified as heterosexual reported same-sex erotic experiences. Second, the surveyed Spanish heterosexual men and women seem to feel discomfort after having any of the evaluated same-sex experiences. In particular, based on our findings, general discomfort with one’s heterosexuality is associated with being younger and male, having a lower level of education, and engaging in same-sex aesthetic-erotic experiences.

As expected by Hypothesis 1, Spanish heterosexual women show a greater interest in the same sex, as has been demonstrated in populations from other countries (Vrangalova & Savin-Williams, 2010). A higher percentage of these women, in comparison to their male counterparts, reported having had same-sex experiences and a greater intention to perform such behaviors. This trend was observed in all variables, except for same-sex relationships, where the difference was not significant. Similarly, these Spanish heterosexual women were also placed, in comparison to Spanish heterosexual men, in intermediate positions on Kinsey’s sexual attraction scale (Copen et al., 2016).

This openness toward experiencing same-sex relations among heterosexual women is in line with the extant literature, which has generally found that women, regardless their sexual orientation, have higher levels of same-sex attraction (Copen et al., 2016; Morales-Knight & Hope, 2012; Vrangalova & Savin-Williams, 2012) and fantasies (Morales-Knight & Hope, 2012) and a greater tendency to identify as bisexual or to be placed in intermediate positions on the Kinsey Scale (Ballester-Arnal & Gil-Llario, 2016; Copen et al., 2016; Gates, 2011; Petersen & Hyde, 2011; Vrangalova & Savin-Williams, 2012). In particular, this trend occurs among women who self-identify as heterosexual (Vrangalova & Savin-Williams, 2010).

One possible explanation for these results could be the heteronormative framework in society (Davis-Delano & Morgan, 2016; Herz & Johansson, 2015), which may exert greater pressure on Spanish men by punishing same-sex behaviors more among them than among Spanish women, as has been observed in other countries (Katz-Wise & Hyde, 2012). These differences are also seen in tolerance toward different sexual orientations. Heterosexual women show the same tolerance toward gay men and lesbian women, whereas heterosexual men show a more positive attitude toward lesbian women than toward gay men (Petersen & Hyde, 2011), possibly because lesbianism has been eroticized by heterosexual men for many years (Louderback & Whitley Jr, 1997). Esterline and Galupo (2013) found that heterosexual women are more often asked to engage in same-sex sexual behavior than heterosexual men. This may point to the prevalence of greater sexual fluidity among women (Diamond, 2008; Katz-Wise, 2015), as in certain specific circumstances, women may feel desire for people of the same sex, regardless of their sexual orientation. Most likely, Spanish heterosexual women question their sexual orientation more than Spanish heterosexual men, as has been reported in other cultures (Morgan & Thompson, 2011; Morgan et al., 2010). Consequently, they may have a greater intention to explore sexual behaviors (such as kissing and caressing) with other women. Heterosexual men are more likely to ask or encourage others to engage in these behaviors than heterosexual women, possibly to fulfill their lesbian fantasies (Yost & McCarthy, 2012).

Regarding erotic experiences with same-sex people, surveyed Spanish heterosexual women showed a greater gap between those few who had engaged in sexual relations and those quite who reported having sexual desires. This gap was lower among surveyed Spanish heterosexual men. In the Spanish culture and other cultures, female sexuality has traditionally been repressed, silenced (Freixas & Luque, 2009; Van Ness et al., 2017), and limited to sexual behaviors that heterosexual men would like women to perform to satisfy their sexual desires and achieve their own pleasure (Emmerink et al., 2016; Lamb, 2010). This lower index of same-sex contact may be related to the lack of behavioral motivation on account of both their own decisions and cultural pressure. Spanish society assumes that women have low interest in sex (Freixas & Luque, 2009); thus, those who show an active interest and actively engage in sexual behavior to seek their own sexual pleasure are often socially punished (Emmerink et al., 2016; Van Ness et al., 2017). This would support the fact that more Spanish heterosexual men fulfill their same-sex sexual desires compared to Spanish heterosexual women.

Regarding discomfort, the greatest percentages are located in engaging in same-sex intercourse and having homoerotic dreams. The first may cause more discomfort because it is the most explicit behavior and entails greater intimate and social exposure. The discomfort around erotica may be associated with the involuntary nature of the dreams themselves and because they are manifestations of latent motivations and desires of the unconscious (Yu & Fu, 2011). The same-sex aesthetic-erotic experience in the heterosexual population produces important figures of discomfort, possibly because of a discrepancy between what they expect of themselves as heterosexuals and their own real experiences (Priolo et al., 2016). In this discomfort, there may be an influence of the rigidity of sexual labels and heterosexuality (Davis-Delano & Morgan, 2016; Davis-Delano et al., 2018). In contrast, in this study, there is a clear diversity within heterosexuality.

Answering RQ1, the general discomfort with one’s sexual orientation is related, in part, to being younger and male and having a lower level of education and aesthetic-erotic experiences related to same-sex individuals. The presence of greater discomfort among heterosexual young people may seem surprising given that they show greater tolerance and acceptance toward homosexuality than older people (Glick & Golden, 2010). However, despite exhibiting a greater level of acceptance of other people’s homosexuality, it may be more difficult for heterosexual youth to accept their own homosexual behaviors. Rowen and Malcolm (2003) found that homosexuals who were in the initial stages of identity formation showed greater levels of internalized homophobia. The development and consolidation of their sexual orientation identity occurs in their adolescence and youth (Morgan, 2013). Thus, these same-sex experiences can put heterosexual young people in a place of doubt, thereby forcing them to reflect on their sexual orientation and causing them discomfort. Conversely, a higher educational level is related to greater tolerance toward homosexuality (Takács & Szalma, 2011). Finally, same-sex experiences can destabilize the self-concept around heterosexuality and generate doubts around their identity, which, as the literature shows (Borgogna et al., 2019; Shearer et al., 2016), is related to greater feelings of discomfort. In any case, other variables that could affect this discomfort should be studied. For example, in this study, the presence of religious beliefs was taken into account, but not the type of religion. In several studies, it has been observed that Muslims show lower levels of tolerance toward homosexuality than Christians (Roggemans et al., 2015; Xie, & Peng, 2018). Similarly, people with traditional gender roles show more negative attitudes toward homosexual people (Ioverno et al., 2019). Such attitudes can generate the rejection of one’s own same-sex behaviors and enhance one’s feelings of discomfort, that is, internalized homophobia (Shidlo, 1994).

Gender is also related to the discomfort associated with different same-sex experiences, with surveyed Spanish heterosexual men showing a higher percentage than Spanish heterosexual women. This may be the result of greater punishments for same-sex behaviors among men (Herek, 2009). At the same time, it can be linked to the rigidity of gender roles of the Spanish culture, the sense of masculinity, and the rejection of femininity (Cornejo, 2015; Parrott, 2009; Parrott & Zeichner, 2008), as heterosexual men associate masculine homosexuality with the feminine and a threat to their own masculinity (Carnaghi et al., 2011; Glick et al., 2007). Badinter (1993) argued that male identity is based on a triple negation, namely, not being a child, not being a woman, and not being gay. In line with Ravenhill and Visser (2018), in contemporary Western societies such as the Spanish society, hegemonic masculinity, a system of beliefs and values, postulates that masculine and dominant men stand at the top of the social hierarchy, whereas women and other men are subordinate, and everything feminine is rejected or undervalued (Connell & Messerschmidt, 2005). Therefore, homosexual men are left out of this hegemonic masculinity as they are associated with femininity. In this sense, homosexuality among men is experienced as an attack on male supremacy in Spanish society. This can explain why heterosexual men present higher levels of homophobia toward gay men and behaviors (Petersen & Hyde, 2011); even in contexts such as the Spanish context, this discrimination manifests itself in violence. To them, it is a strategy to get away from everything related to femininity and being gay. Among gay men, not all are considered equal in society. There is a social hierarchy whereby “top” men (insertive in anal sex) are perceived as more masculine and “bottom” men (receptive in anal sex) are perceived as less masculine (Ravenhill & de Visser, 2017). In some Latin American and Arabic societies, the “top” man is not considered homosexual or undervalued but is rather seen as more masculine and dominant (Carroll, 2015). Being anally penetrated is a clear example of “subordination” and “weakness” in a man. Accordingly, heterosexual men are least willing to carry out this behavior.

These results should be considered in light of a possible social desirability based on self-reportage, especially regarding issues that may go against one’s moral values or social norms, such as sexuality and sexual orientation (Sutton, 2016). Second, we must be cautious while generalizing some of these results because the sample was obtained through convenience sampling. Therefore, these findings should help future research be more representative. Third, this study was advertised as an investigation of sexual orientation. Thus, heterosexual people who agreed to participate may have been more open-minded than the general population, and the results may have thus been skewed. The closed nature of the measures used, such as dichotomous variables, may have also limited the interpretation of the results. Future research may consider extending the age of the sample and including more heterosexual men to obtain a less unbalanced sample by gender.

### Conclusions

Our findings offer valuable information about sexual diversity among heterosexuals, which is often accompanied by significant levels of discomfort regarding different sexual manifestations. This may limit the experience, enjoyment, and full development of sexuality (Greenberg et al., 2017), especially among heterosexual men. These findings were specific to Spain, which is a country where legislative advances coexist with an unshakable religious and machismo tradition. These data should be taken into account while carrying out therapeutic and sexual education programs aimed at working on self-acceptance and tolerance and publicizing how diverse society and sexuality can be. It is clear that there is a need to continue studying sexual orientation as a complex construct to include the affective-sexual diversity that characterizes the population in its entirety.

## References

[CR1] Abou-Chadi T, Finnigan R (2019). Rights for same-sex couples and public attitudes toward gays and lesbians in Europe. Comparative Political Studies.

[CR2] Ajzen I (1991). The theory of planned behavior. Organizational Behavior and Human Decision Processes.

[CR3] Badinter, E. (1993). *XY. La identidad masculina* [XY. The male identity]. Alianza Editorial.

[CR4] Ballester-Arnal, R., & Gil-Llario, M. D. (2016). Orientación del deseo sexual: Crisis y decadencia de los modelos categoriales [Orientation of sexual desire: Crisis and decline of the categorical models]. In A. López de la Llave (Ed.), *Sexología positiva: Placer, salud y bienestar* (pp. 59-66). Universidad Nacional de Educación a Distancia.

[CR5] Birkett M, Espelage DL, Koenig B (2009). LGB and questioning students in schools: The moderating effects of homophobic bullying and school climate on negative outcomes. Journal of Youth and Adolescence.

[CR6] Borgogna NC, McDermott RC, Aita SL, Kridel MM (2019). Anxiety and depression across gender and sexual minorities: Implications for transgender, gender nonconforming, pansexual, demisexual, asexual, queer, and questioning individuals. Psychology of Sexual Orientation and Gender Diversity.

[CR7] Carnaghi A, Maass A, Fasoli F (2011). Enhancing masculinity by slandering homosexuals: The role of homophobic epithets in heterosexual gender identity. Personality and Social Psychology Bulletin.

[CR8] Carroll JL (2015). Sexuality now: Embracing diversity.

[CR9] Connell RW, Messerschmidt JW (2005). Hegemonic masculinity: Rethinking the concept. Gender & Society.

[CR10] Copen, C. E., Chandra, A., & Febo-Vazquez, I. (2016). Sexual behavior, sexual attraction, and sexual orientation among adults aged 18–44 in the United States: Data from the 2011–2013 National Survey of Family Growth. *National Health Statistics Reports (No. 88)*. Retrieved July 13, 2019, from https://www.cdc.gov/nchs//data/nhsr/nhsr088.pdf 26766410

[CR11] Cornejo, J. (2015). Componentes ideológicos de la homofobia [Ideological components of homophobia]. *Límite. Revista de Filosofía y Psicología*, *7*(26), 85–106.

[CR12] Davis-Delano LR, Morgan EM (2016). Heterosexual identity management: How social context affects heterosexual marking practices. Identity.

[CR13] Davis-Delano LR, Morgan EM, Gillard A, Davis CV (2018). When heterosexuality is questioned: Stifling suspicion through public displays of heterosexual identity. Journal of Homosexuality.

[CR14] Diamond LM (2008). Sexual fluidity: Understanding women’s love and desire.

[CR15] Eisner S (2013). Bi: Notes for a bisexual revolution.

[CR16] Emmerink PM, Vanwesenbeeck I, van den Eijnden RJ, ter Bogt TF (2016). Psychosexual correlates of sexual double standard endorsement in adolescent sexuality. Journal of Sex Research.

[CR17] Esterline KM, Galupo MP (2013). ‘Drunken curiosity’ and ‘gay chicken’: Gender differences in same-sex performativity. Journal of Bisexuality.

[CR18] European Union Agency for Fundamental Rights. (2020). *A long way to go for LGBTI equality.* Retrieved September 15, 2020, from https://fra.europa.eu/sites/default/files/fra_uploads/fra-2020-lgbti-equality-1_en.pdf

[CR19] Freixas, A., & Luque, B. (2009). El secreto mejor guardado: La sexualidad de las mujeres mayores [The best-kept secret: Older women's sexuality]. *Revista Política y Sociedad*, *46*(1–2), 191–203.

[CR20] Gates, G. (2011). *How many people are lesbian, gay, bisexual, and transgender?* Retrieved July 25, 2019, from http://williamsinstitute.law.ucla.edu/wp-content/uploads/Gates-How-Many-People-LGBT-Apr-2011.pdf

[CR21] Gil-Llario MD, Giménez C, Ballester-Arnal R, Cárdenas-López G, Durán-Baca X (2017). Gender, sexuality, and relationships in young Hispanic people. Journal of Sex & Marital Therapy.

[CR22] Giménez-García C, Castro-Calvo J, Gil-Llario MD, Ballester-Arnal R (2020). Sexual relationships in Hispanic countries: A literature review. Current Sexual Health Reports.

[CR24] Glick SN, Golden MR (2010). Persistence of racial differences in attitudes toward homosexuality in the United States. Journal of Acquired Immune Deficiency Syndromes.

[CR23] Glick P, Gangl C, Gibb S, Klumpner S, Weinberg E (2007). Defensive reactions to masculinity threat: More negative affect toward effeminate (but not masculine) gay men. Sex Roles.

[CR25] Greenberg JM, Smith KP, Kim TY, Naghdechi L, IsHak WW, IsHak W (2017). Sex and quality of life. The textbook of clinical sexual medicine.

[CR26] Hayfield N (2020). Bisexual and pansexual identities: Exploring and challenging invisibility and invalidation.

[CR27] Herek GM (2009). Hate crimes and stigma-related experiences among sexual minority adults in the United States: Prevalence estimates from a national probability sample. Journal of Interpersonal Violence.

[CR28] Herz M, Johansson T (2015). The normativity of the concept of heteronormativity. Journal of Homosexuality.

[CR29] Hogg MA, Yzerbyt V, Judd CM, Corneille O (2004). Uncertainty and extremism: Identification with high entitativity groups under conditions of uncertainty. The psychology of group perception: Perceived variability, entitativity, and essentialism.

[CR30] International Lesbian, Gay, Bisexual, Trans and Intersex Association. (2020). *State-sponsored homophobia 2020: Global legislation overview update.* Retrieved January 10, 2021, from https://ilga.org/downloads/ILGA_World_State_Sponsored_Homophobia_report_global_legislation_overview_update_December_2020.pdf

[CR31] Ioverno S, Baiocco R, Lingiardi V, Verrastro V, D’Amore S, Green RJ (2019). Attitudes towards same-sex parenting in Italy: The influence of traditional gender ideology. Culture, Health & Sexuality.

[CR32] Kalfon P, Mimoz O, Auquier P, Loundou A, Gauzit R, Lepape A, Laurens J, Garrigues B, Pottecher T, Mallédant Y (2010). Development and validation of a questionnaire for quantitative assessment of perceived discomforts in critically ill patients. Intensive Care Medicine.

[CR33] Katz-Wise SL (2015). Sexual fluidity in young adult women and men: Associations with sexual orientation and sexual identity development. Psychology & Sexuality.

[CR34] Katz-Wise SL, Hyde JS (2012). Victimization experiences of lesbian, gay, and bisexual individuals: A meta-analysis. Journal of Sex Research.

[CR35] Kinsey AC, Pomeroy WB, Martin CE (1948). Sexual behavior in the human male.

[CR36] Kite ME, Togans LJ, Schultz TJ, Keith KD (2019). Stability or change? A cross-cultural look at attitudes toward sexual and gender identity minorities. Cross-cultural psychology: Contemporary themes and perspectives.

[CR37] Klein F (1978). The bisexual option.

[CR38] Krueger EA, Meyer IH, Upchurch DM (2018). Sexual orientation group differences in perceived stress and depressive symptoms among young adults in the United States. LGBT Health.

[CR39] Lamb S (2010). Feminist ideals for a healthy female adolescent sexuality: A critique. Sex Roles.

[CR40] Lin CL, Wang MJJ, Drury CG, Chen YS (2010). Evaluation of perceived discomfort in repetitive arm reaching and holding tasks. International Journal of Industrial Ergonomics.

[CR41] Louderback LA, Whitley BE (1997). Perceived erotic value of homosexuality and sex-role attitudes as mediators of sex differences in heterosexual college students' attitudes toward lesbians and gay men. Journal of Sex Research.

[CR42] Milot-Lapointe F, Le Corff Y, Savard R (2019). A study of clinical change in individual career counseling. Career Development Quarterly.

[CR43] Morales-Knight LF, Hope DA (2012). Correlates of same-sex attractions and behaviors among self-identified heterosexual university students. Archives of Sexual Behavior.

[CR44] Morgan EM (2013). Contemporary issues in sexual orientation and identity development in emerging adulthood. Emerging Adulthood.

[CR45] Morgan EM, Thompson EM (2011). Processes of sexual orientation questioning among heterosexual women. Journal of Sex Research.

[CR46] Morgan EM, Steiner MG, Thompson EM (2010). Processes of sexual orientation questioning among heterosexual men. Men and Masculinities.

[CR47] Nebot-Garcia, J. E., Ballester-Arnal, R., & Ruiz-Palomino, E. (2020). Atracción, deseos y conductas sexuales: Evidencias de la diversidad en la orientación sexual de jóvenes españoles [Sexual attraction, desires and behaviors: Evidence of diversity in sexual orientation among Spanish youth]. *Informació Psicológica*, (120), 20–34. 10.14635/IPSIC.2020.120.1

[CR48] Nebot-Garcia, J. E., García-Barba, M., Gil-Juliá, B., Giménez-García, C., & Ballester-Arnal, R. (2018). Comportamientos homosexuales en jóvenes heterosexuales: Diferencias de género [Homosexual behaviors in young heterosexuals: Gender differences]. *Àgora de Salut*, *5*, 59–68. 10.6035/AgoraSalut.2018.5.6

[CR49] Parrott DJ (2009). Aggression toward gay men as gender role enforcement: Effects of male role norms, sexual prejudice, and masculine gender role stress. Journal of Personality.

[CR50] Parrott DJ, Zeichner A (2008). Determinants of anger and physical aggression based on sexual orientation: An experimental examination of hypermasculinity and exposure to male gender role violations. Archives of Sexual Behavior.

[CR51] Petersen JL, Hyde JS (2011). Gender differences in sexual attitudes and behaviors: A review of meta-analytic results and large datasets. Journal of Sex Research.

[CR52] Pichardo JI (2011). We are family (or not): Social and legal recognition of same-sex relationships and lesbian and gay families in Spain. Sexualities.

[CR53] Priolo D, Milhabet I, Codou O, Fointiat V, Lebarbenchon E, Gabarrot F (2016). Encouraging ecological behaviour through induced hypocrisy and inconsistency. Journal of Environmental Psychology.

[CR54] Rama J, Zanotti L, Turnbull-Dugarte SJ, Santana A (2021). VOX: The rise of the Spanish populist radical right.

[CR55] Rault W, Digoix M (2020). Postface. After Legal Recognition. Same-sex families and legal recognition in Europe. European studies of population.

[CR56] Ravenhill JP, de Visser RO (2017). Perceptions of gay men’s masculinity are associated with their sexual self-label, voice quality and physique. Psychology & Sexuality.

[CR57] Ravenhill JP, de Visser RO (2018). “It takes a man to put me on the bottom”: Gay men’s experiences of masculinity and anal intercourse. Journal of Sex Research.

[CR58] Rodríguez-Temiño I, Almansa-Sánchez J (2021). The use of past events as political symbols in Spain. The example of Vox and the need for a new archaeology of ethnicity. International Journal of Heritage Studies.

[CR59] Roggemans L, Spruyt B, Droogenbroeck FV, Keppens G (2015). Religion and negative attitudes towards homosexuals: An analysis of urban young people and their attitudes towards homosexuality. Young.

[CR60] Rose D, Ussher JM, Perz J (2017). Let's talk about gay sex: Gay and bisexual men's sexual communication with healthcare professionals after prostate cancer. European Journal of Cancer Care.

[CR61] Rowen CJ, Malcolm JP (2003). Correlates of internalized homophobia and homosexual identity formation in a sample of gay men. Journal of Homosexuality.

[CR62] Santos AC, Santos AC (2013). Political, legal and cultural change in Southern Europe. Social movements and sexual citizenship in southern Europe.

[CR63] Scroggs B, Durtschi J, Busk M, Goodcase E, Jones DL (2020). Within-minority group discomfort in lesbian, gay, and bisexual emerging adults of color: Implications for group identification and well-being. Journal of Gay & Lesbian Mental Health.

[CR64] Shearer A, Herres J, Kodish T, Squitieri H, James K, Russon J, Atte T, Diamond GS (2016). Differences in mental health symptoms across lesbian, gay, bisexual, and questioning youth in primary care settings. Journal of Adolescent Health.

[CR65] Shidlo A, Greene B, Herek GM (1994). Internalized homophobia: Conceptual and empirical issues in measurement. Psychological perspectives on lesbian and gay issues, Vol. 1. Lesbian and gay psychology: Theory, research, and clinical applications.

[CR66] Sutton GW (2016). A house divided: Sexuality, morality, and Christian cultures.

[CR67] Takács J, Szalma I (2011). Homophobia and same-sex partnership legislation in Europe. Equality, Diversity and Inclusion: An International Journal.

[CR68] Van Ness N, Miller MM, Negash S, Morgan M (2017). Embracing our eroticism: A Foucauldian discourse analysis of women’s eroticism. Journal of Feminist Family Therapy.

[CR69] Vrangalova Z, Savin-Williams RC (2010). Correlates of same-sex sexuality in heterosexually identified young adults. Journal of Sex Research.

[CR70] Vrangalova Z, Savin-Williams RC (2012). Mostly heterosexual and mostly gay/lesbian: Evidence for new sexual orientation identities. Archives of Sexual Behavior.

[CR71] Xie Y, Peng M (2018). Attitudes toward homosexuality in China: Exploring the effects of religion, modernizing factors, and traditional culture. Journal of Homosexuality.

[CR72] Yost MR, McCarthy L (2012). Girls gone wild? Heterosexual women’s same-sex encounters at college parties. Psychology of Women Quarterly.

[CR73] Yu CKC, Fu W (2011). Sex dreams, wet dreams, and nocturnal emissions. Dreaming.

